# Hybrid recurrent with spiking neural network model for enhanced anomaly prediction in IoT networks security

**DOI:** 10.3389/frai.2025.1651516

**Published:** 2025-10-09

**Authors:** Mohammed Mustafa, Sarah M. Eljack Babiker, Yasir Eltigani Ali Mustafa

**Affiliations:** ^1^Faculty of Computers and Information Technology, University of Tabuk, Tabuk, Saudi Arabia; ^2^Department of Computer Science and Information, College of Science, Majmaah University, Al-Majmaah, Saudi Arabia; ^3^Department of Computer Science and Information Technology, Ahmed bin Mohammed Military College, Doha, Qatar

**Keywords:** IoT security, intrusion detection system (IDS), recurrent neural networks, spiking neural network, SMOTE, recursive feature elimination

## Abstract

**Introduction:**

As the number of Internet of Things (IoT) devices grows quickly, cyber threats are becoming more complex and increasingly sophisticated; thus, we need a more robust network security solutions. Traditional deep learning approaches often suffer in identifying effectively anomalies in IoT network. To tackle this evolving challenge, this research proposes a hybrid architecture of Neural Network (NN) models that combine Recurrent-NN (RNN) and Spiking-NN (SNN), referred to as HRSNN, to improve IoT the security.

**Methods:**

The proposed HRSNN technique has five steps: preprocessing data, extracting features, equalization classes, features optimization and classification. Data processing step makes sure that input data is accurate and consistent and by employing normalization and the removal of outliers’ techniques. Feature extraction makes use of the RNN part to automatically detect abnormal patterns and high-level features, which are then turned into spike trains for the SNN to process over time. In class equalization step, the Synthetic Minority-Oversampling Technique (SMOTE) is being used resulting in balanced classes. Recursive Feature Elimination (RFE) is used to keep the important features for feature optimization. Then, the dataset is split into sets for testing and training so that the model can be tested properly.

**Results:**

The hybrid model integrates the spatial feature learning skills of RNNs with the temporal adaptability of SNNs, results in an improved accuracy and resilience in identifying IoT network abnormalities. The proposed HRSNN approach, which was tested on the CIC-IoT23 and TON_IoT data sets, achieved better performance compared to current deep learning (DL) models. In particular, experimental assessments show that the model attained an accuracy rate of 99.5% on the “CICIoT2023” dataset and 98.75% on the “TON_IoT” dataset.

**Discussion:**

These results confirm demonstrate that the proposed architecture of RNN and SSN can achieve significant advancement to IoT security. By combining both spatial and temporal feature learning, HRSNN can improve accuracy detection against diverse security threats. The model is reliable, accurate, and adaptable for safeguarding IoT networks against diverse security threats. Thus, the model addresses the potential solutions in the challenging problem of secured IoT networks.

## 1 Introduction

The rapid evolution of IoT, cloud computing, and cybersecurity technologies has emerged in a new era of interconnected systems. IoT, with its potential to revolutionize various sectors like smart homes, industries, and cities, offers enhanced adaptability and productivity. By enhancing flexibility and productivity, IoT evolves the creation of highly interconnected systems that support innovative services (Zaman et al., 2021). These benefits make IoT suitable for both commercial and industrial use cases. The IoT’s evolution has also coincided with the evolution of specialized solutions, shaping the structure of Industrial IoT (IIoT) and Industry 4.0 over the past decade (Alabadi et al., 2022). Projections suggest that by 2030, the number of IoT devices worldwide will triple from the current 15.14 billion. Approximately 60% of these devices are utilized in commercial markets and business sectors, a trend predicted to remain consistent over the next decade (Choudhary, 2024). However, the inherent transparency and dynamic nature of IoT networks, integrated with the resource constraints of IoT devices, makes them highly susceptible to cyberattacks.

**Figure 1:**
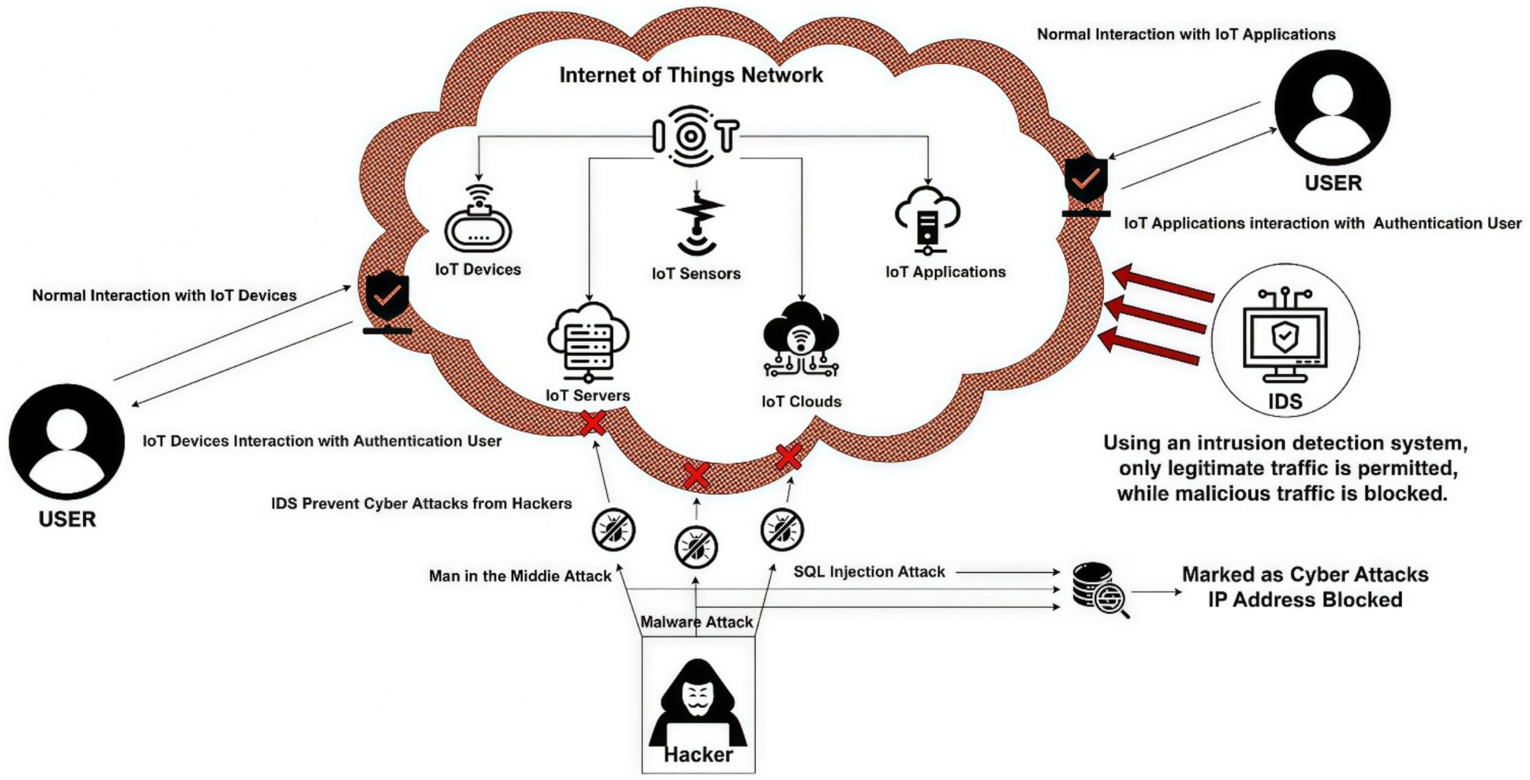
Intrusion detection system (IDS)-based security in IoT outline.

Figure 1 outlines the architecture of an IoT network, comprising devices, cloud services, actuators, servers, sensors, protocols, and applications, and illustrates a framework for anomaly-based IDS in IoT networks (Bakhsh et al., 2023). These elements work with both authorized and unauthorized users, which makes it hard to find the difference between regular behavior and malicious behavior. Unauthorized users can take advantage of vulnerabilities in security systems, which can lead to cyberattacks. As the number of IoT devices in homes and businesses increases, the likelihood of cyberattacks also rises. Because these devices do not have enough memory, processing capacity, or security, they are easy targets for hackers. Hackers take advantage of vulnerabilities to build botnets, interrupt services, private information leakage, and invade users’ privacy. Strong security measures are required to minimize these threats. VPNs and other secure authentication and encrypted connection methods keep data safe and private.

IDS make security even better by monitoring and finding abnormal behaviors. Signature-based IDS (SIDS) is one type of IDS that tracks for known threats, while Anomaly-based IDS (AIDS) is another type that tracks for unknown or abnormal behaviors. As attacks get more complicated and IoT devices cannot handle as much data, traditional security solutions will encounter difficulties with new threats. DL and machine learning (ML) have become viable options to make IoT networks more secure. These artificial intelligence (AI)-powered methods can detect and prevent attacks by analyzing patterns in network data and find unusual behaviors. AI has evolved better, making DL a better algorithm for IoT-IDS.

Deep Neural Networks (DNN) are effective in handling large volume of data, finding patterns, and sorting data. These systems are great for AIDS, since they track on servers, clouds, sensors, and devices to keep IoT infrastructure safe. Suricata and Snort are two examples of IDS that can help to minimize the damage caused by botnet attacks. These systems use analytical methods to analyze how malware behaves in certain settings (Lifi et al., 2023; Alkhonaini et al., 2024). DL and ML approaches require this behavioral information since they need to keep collecting data while the virus is functioning. These approaches show how malware can affect a system and cause problems (Alrefaei and Illyas, 2024; Nazir et al., 2024).

Techniques for classification that have been trained on previous attacks can find threats more easily. For instance, once an IoT botnet carries out a Distributed-Denial-of-Services (DDoS) attack, the model may learn to identify other DDoS threats and botnet behaviors in similar settings (Saurabh et al., 2025). AI is being used more to find IoT intrusions because it can adapt to new attack patterns due to its enhanced detection skills (Ayad et al., 2024; Saurabh et al., 2024). But there are still problems since attackers are advancing with new versions of old techniques that are hard for current solutions to detect. Recent studies have targeted on developing AI techniques to improve the detection of threats in IoT settings (Asgharzadeh et al., 2024; Gelgi et al., 2024) to solve these problems. Adding DL and ML models to secure devices have been shown to make them work better and be more flexible. Hybrid AI methods, such as combining genetic algorithms (GA) with DL, have shown great potential for finding problems in IoT networks by improving the selection of features and accuracy of models (Almuqren et al., 2023; Hanafi et al., 2024). DL is one of the most advanced AI methods since it can handle complicated, nonlinear data. It has been used effectively in many real-world situations. Researchers are always working to make DL models better so that IDS works better. These advancements offer a promising approach to address IoT security challenges and protect against evolving threats. This research aims to develop an IDS with a hybrid NN methodology for identifying diverse classes of attacks generally noticed in IoTs. These attacks include HTTP Flood, Bruteforce, TCP Flood, DoS, and UDP Flood.

As the hybrid DL models like LSTM-CNN and BiGRU-CNN suffering from increased computational complexity, this research proposes the HRSNN model which includes the benefits of RNN and SNN in a complementary manner. The RNN part is responsible for capturing spatial and sequential relations in IoT traffic, while the temporal spike-based encoding of SNN, which resembles neuromorphic processing, allows for more energy-efficient and event-driven processing. This combination improves anomaly detection and eliminates unnecessary computation. This makes it ideal for IoT devices with limited resources, such as memory, power, and latency. HRSNN combines biological plausibility along with DL to strike an effective balance of performance and efficiency that is not achievable by existing deep hybrid networks, which makes it an innovative and valuable solution for IoT intrusion detection. The primary contributions of the proposed research are described in the following:

- The study introduces an HRSNN model for IoT network security, combining RNN’s spatial feature extraction with SNN’s temporal adaptability for robust anomaly detection.
- Key contributions include a five-stage data processing pipeline, class equalization using SMOTE, and feature optimization via RFE.
- Superior performance on the CIC-IoT23 and TON_IoT datasets, achieving enhanced accuracy, reduced false positives, and computational efficiency, making it a scalable and practical solution for IoT security.

The article is structured in the following manner: Section 2 analyzes the security challenges of IoT and DL applications in IDS with current models. Section 3 details the developed IDS model, including its architectures and mathematical formulations. Section 4 discusses evaluation metrics, experimental results, and comparative analysis with current conventional ML and DL-based IDS models. Section 5 concludes with key findings.

## 2 Literature review

This section highlights the need for robust cybersecurity in the rapidly expanding IoT ecosystem. It reviews existing research on leveraging DL and ML techniques to design intrusion detection for IoT. The problems that need to be solved in IoT-IDS include, efficient solutions with resources, dealing with data scarcity and asymmetry, and getting real-time performance. It also points out important areas of research that need to be filled, such as making AI models that can be explained and evolve with the ever-changing threat landscape.

Asgharzadeh et al. (2024) proposed a methodology for identifying anomalies in IoT networks through the development of a specialized Convolutional Neural Network (FECNNIoT) for enhanced feature extraction. The approach used a better optimization algorithm called the Binary Multi-objectives Enhanced Gorilla Troop Optimization (BMEGTO) to choose the best features. A (K-Nearest Neighbors (KNN) algorithm then analyzed the improved feature set to find intrusions with a high level of accuracy. The CNN-BMEGTO-KNN framework was highly accurate with 99.99% on the TON-IoT and 99.86% on the NSL-KDD data sets. It also reduced the feature set down to 27 and 25% of the initial features.

Hanafi et al. (2024) developed a new IDS for IoT networks that used a Long Short-Term Memory (LSTM) network and an Improved Binary Golden Jackal Optimizer (IBGJO) method. The IBGJO method, improved by Opposition-Based Learning, quickly chose the most relevant features from the data set, which made the classification more accurate. The LSTM network then used these optimized features to accurately classify data, achieving an excellent rate of detection of 98.21% on both the CICIDS 2017 and NSL-KDD datasets. The model outperformed current approaches, including BGJO-LSTM, BWOA-LSTM, BSCA-LSTM, SVM, KNN, and Naive Bayes, showing that it is better at finding intrusions in IoT networks.

Kethineni and Pradeepinni (2024) presented a DL-based architecture for intrusion detection in smart agricultural systems. The three-tier design included a fog processing layer for networked computing. It also combined the CNN with a Bidirectional Gated Recurrent Unit (CNN-BiGRU) model that had an attention mechanism to find DDoS attacks. The Wild Horse Optimization technique was used to make the model work better. It achieved high accuracy rates of 99.35% on APA-DDoS and 99.71% on ToN-IoT datasets

Yaras and Denner (2024) concentrated on improving the detection of attacks in IoT networks by the implementation of a hybrid DL model that included 1D-CNN and LSTM. The technique was developed and assessed using big data tools such as Apache Spark and PySpark within the Google Collaboratory. It analyzed the network data to find cyberattacks, especially DDoS attacks, with an accuracy of 99.995% on CICIoT2023 and 98.75% on TON_IoT. This method worked well to solve the problems of analyzing big data sets made by IoT networks, giving a strong way to improve security.

Qaddos et al. (2024) developed a hybrid DL model for IoT security detection, integrating CNN and GRU to proficiently capture intricate characteristics and temporal correlations in IoT data. To deal with the imbalance in data, the Feature-Weighted SMOTE (FW-SMOTE) method was included, and the model was tested on the IoTID20 and UNSW-NB15 data sets. The model performed better than current benchmarks, with accuracy rates of 99.60 and 99.16%. These results showed that the model worked well and could be changed to fit different needs. This is a viable solution to make IoT ecosystems more secure and resilient against changing cyber threats.

Kasongo and Sun (2019) proposed an IDS system employing RNN architectures (LSTM, GRU, Simple RNN) for detecting attacks on the NSL-KDD and UNSW-NB15 datasets. A feature selection technique based on XGBoost minimized the set of features to only the useful ones. The XGBoost-LSTM model performed better in classifying NSL-KDD data into two groups (TAC: 88.13%, VAC: 99.49%). The XGBoost-Simple-RNN model performed better in classifying UNSW-NB15 data into two groups (TAC: 87.07%). XGBoost-LSTM (NSL-KDD) and XGBoost-GRU (UNSW-NB15) were the most accurate for classifying multiclass attacks. By integrating feature selection with the RNN architectures, the framework made detection of attacks better.

Hizal et al. (2024) worked on making intrusion detection better in IoT networks, with an emphasis on DDoS attacks. It used a two-level DL-based IDS and included preprocessing methods like feature reduction and data adjustment to make the model work better. The study demonstrated that a two-stage strategy, assessed using fully connected, combined, and LSTM models on the CICIoT2023 dataset, surpassed conventional single-model techniques such as DNN, CNN, and RNN, providing superior and more resilient detection of DDoS attacks in IoT networks.

Wang et al. (2024) worked on making IoT networks safer by building a new IDS. The system employed a form of DL called Conditional Tabular Generative Adversarial Networks (CTGAN) to fix the problem of unbalanced data, where some attacks were less prevalent. The system performed better in finding other sorts of intrusions by making fake data to identify these less common attempts. The model was evaluated on number of datasets and attained a higher accuracy compared to current approaches.

Sajid et al. (2024) developed a new hybrid IDS model that used XGBoost and CNN for the extraction of features and LSTM for classification. This framework was designed to deal with the increasing difficulty of network security, which includes problems like new sorts of attacks and vast amounts of different types of data. The model used XGBoost and CNN to choose features and LSTM to classify them. It had a high detection rate and a low false acceptance rate across the four test datasets (CIC IDS 2017, UNSW NB15, NSL KDD, and WSN DS). This shows that it is effective at improving network security against threats that change over time.

Zia et al. (2024) emphasized the security issues arising from the increasing application of IoTs in cities that are smart cities, especially in zero-touch networks (ZTN) that independently regulate network resources. It created a new DL-based IDS (DL-NIDS-ZTN) that employed CNN to find several types of intrusions, such as DDoS, Botnet, Brute force, and Invasion. The model has a very high accuracy of 99.80% on the CICIDS-2018 dataset, making it a strong way to protect the integration of IoT equipment and services in smart cities.

Recent studies on AI-driven zero-day intrusion detection focus on ML and DL approaches, as well as anomaly detection and hybrid methods. While each of these generally performs well, each one has significant limitations. A large number of studies are limited in scope, concentrating on certain kinds of attacks or certain working environments. This is on top of a set of challenges that continue to exist, such as high processing costs, the need for real-time response, the ability to handle increasing workloads, and the change of working environments. A hybrid approach combining the strengths of multiple algorithms in overcoming the limitations of individual algorithms is a useful trend in the literature. While the studies discussed above are helpful, there is a glaring gap when it comes to addressing zero-day attacks and the need for newer, more efficient AI models (Yee et al., 2024).

In a study by Dai et al. (2024), the CIC-MalMem-2022 data set was used. This study discovered new attacks using auto encoders (AE) for anomaly detection and further analyzed using XGBoost and Random Forest (RF) to create newer hybrid models. The new models that combined anomaly detection and classical ML classifiers had better detection rates, the best being the RF-AE model that had flawless detection. The model performed almost equally, having 99.9892% correct classification showing that the model generalized well Table 1 shows research gap among IDS-based Security Schemes for IoT. This study shows the usefulness of metrics combining anomaly and classification approaches in the insurmountable zero-day intrusion detection problem.

**Table 1:** Research gap among IDS-based security schemes for IoT.

Authors	Method name	Merits	Demerits
Asgharzadeh et al. (2024)	CNN-BMEGTO-KNN	The CNN-BMEGTO-KNN model offers high accuracy (99.99%) and efficient feature reduction, making it suitable for large-scale IoT networks.	Integrating the custom CNN and BMEGTO may increase complexity and computational costs, limiting scalability for resource-constrained devices.
Hanafi et al. (2024)	IBGJO-LSTM	The IBGJO-LSTM model achieves high detection accuracy (98.21%), making it highly effective for intrusion detection in IoT networks.	Combining IBGJO with LSTM can make the system more complex and require more computing power, which may not be ideal for IoT devices with limited resources.
Kethineni and Pradeepinni (2024)	CNN-BiGRU	The CNN-BiGRU model effectively combines the strengths of both convolutional and recurrent networks, achieving high accuracy in detecting DDoS attacks in smart farming systems.	The model’s complexity may require substantial computational resources, making it less suitable for deployment on devices with limited processing power.
Yaras and Denner (2024)	hybrid CNN-LSTM	The hybrid model achieves exceptional accuracy, making it highly effective for detecting DDoS attacks in IoT networks.	The use of big data technologies and complex deep learning models may require a lot of computational power, making it difficult to deploy on IoT devices with limited resources.
Qaddos et al. (2024)	hybrid CNN-GRU	The hybrid CNN-GRU model achieves high accuracy (99.60 and 99.16%), making it highly effective for detecting attacks in an IoT network.	The model’s intricacy and the usage of FW-SMOTE for data maintenance may make it harder to run, which might make it less scalable for IoT devices with limited resources.
Kasongo and Sun (2019)	XGBoost-LSTM	Using XGBoost for picking features and RNN architectures together makes the model more successful by making it more accurate for both binary form and multiple-class intrusion detection tasks.	Using more than one RNN architecture with XGBoost for selecting features might make the model more complicated to run, which would make it less useful for immediate identification on IoT devices with limited resources.
Hizal et al. (2024)	convolutional, and LSTM	The two-stage deep learning approach provides more accurate and robust DDoS attack detection compared to traditional single-model methods.	The two-level approach is more complicated, which may mean it needs more computing power and may not work as well for immediate detection on IoT devices with limited resources.
Wang et al. (2024)	CTGAN	CTGAN improves detection accuracy by generating synthetic data for rare attacks, enhancing the system’s performance.	The use of CTGAN may increase computational costs, making the system less suitable for real-time use on resource-limited IoT devices.
Sajid et al. (2024)	Hybrid XGBoost and CNN for feature selection and using LSTM	The hybrid model effectively combines feature extraction and classification techniques, resulting in high detection accuracy and low False Acceptance Rate across multiple datasets.	The complexity of combining XGBoost, CNN, and LSTM may increase computational requirements, making it less suitable for real-time detection on resource-constrained devices.
Zia et al. (2024)	DL-NIDS-ZTN	The DL-NIDS-ZTN model demonstrates high accuracy (99.80%) in detecting multiple types of network intrusions, enhancing security in smart cities.	The complexity of deep learning models may require significant computational resources, potentially limiting their applicability in resource-constrained IoT environments within smart cities.
Yee et al. (2024)	ML, DL, anomaly detection, and hybrid models combining multiple algorithms.	Provides a structured overview of AI-driven intrusion detection techniques. Highlights the role of hybrid models in addressing weaknesses of individual algorithms. Identifies strengths of ML/DL in detecting previously unseen attacks.	Many reviewed models focus only on specific attack types or environments. Limited real-world adaptability due to computational demands and scalability issues. Lacks empirical validation since it is a review, not an implementation.
Dai et al. (2024)	AE-based anomaly detection integrated with XGBoost-AE, RF-AE.	Hybrid anomaly detection-classification framework enhances detection capability. RF-AE achieved near-perfect results (100% accuracy, precision, recall, F1, MCC). Demonstrated strong generalization to unseen data (99.98% accuracy).	Performance heavily dependent on dataset quality (CIC-MalMem-2022). May face scalability challenges in real-time IoT/enterprise networks. Extremely high accuracy might indicate possible overfitting if not tested on broader datasets.
Sana et al. (2024)	ML models (tree-based SVM, Ensemble Bagged Tree, Random Forest, NN) and DL (LSTM, Vision Transformer) with Bayesian optimization.	RF and Ensemble Bagged Tree achieved >99.9% accuracy with AUC = 1.00. LSTM achieved 99.97% accuracy. ViT architecture achieved perfect training metrics and outperformed others. Demonstrated a three-pronged improvement: reliability, security, and network performance.	ViT achieved perfect training accuracy but only 78.70% validation accuracy, suggesting potential overfitting and weaker generalization. High computational cost of ViT may limit deployment on resource-constrained IoT devices. Requires careful optimization for practical real-time scenarios.

The research by Sana et al. (2024) developed the anomaly detection methods in IoT intrusion detection through the merging of advanced IoT ML models and DL models. The special tuning of the model parameters, that is, the Bayesian optimization approach, was very successful in the fine-tuning of the model parameters. This study shows the successful merging of classical IoT models and DL models as a step forward in intrusion detection, giving better attack mitigation, improved safety, and better IoT network operation.

It was stated that IDS models can track network traffic and find DDoS attacks, and that ML and DL models to perform tasks effectively. The system used the accuracy, f-measure, and other metrics to test the proposed hybrid strategy, which combines SNN and RNN and compared it to other standard ML and DL methods.

## 3 Proposed methodology

**Figure 2:**
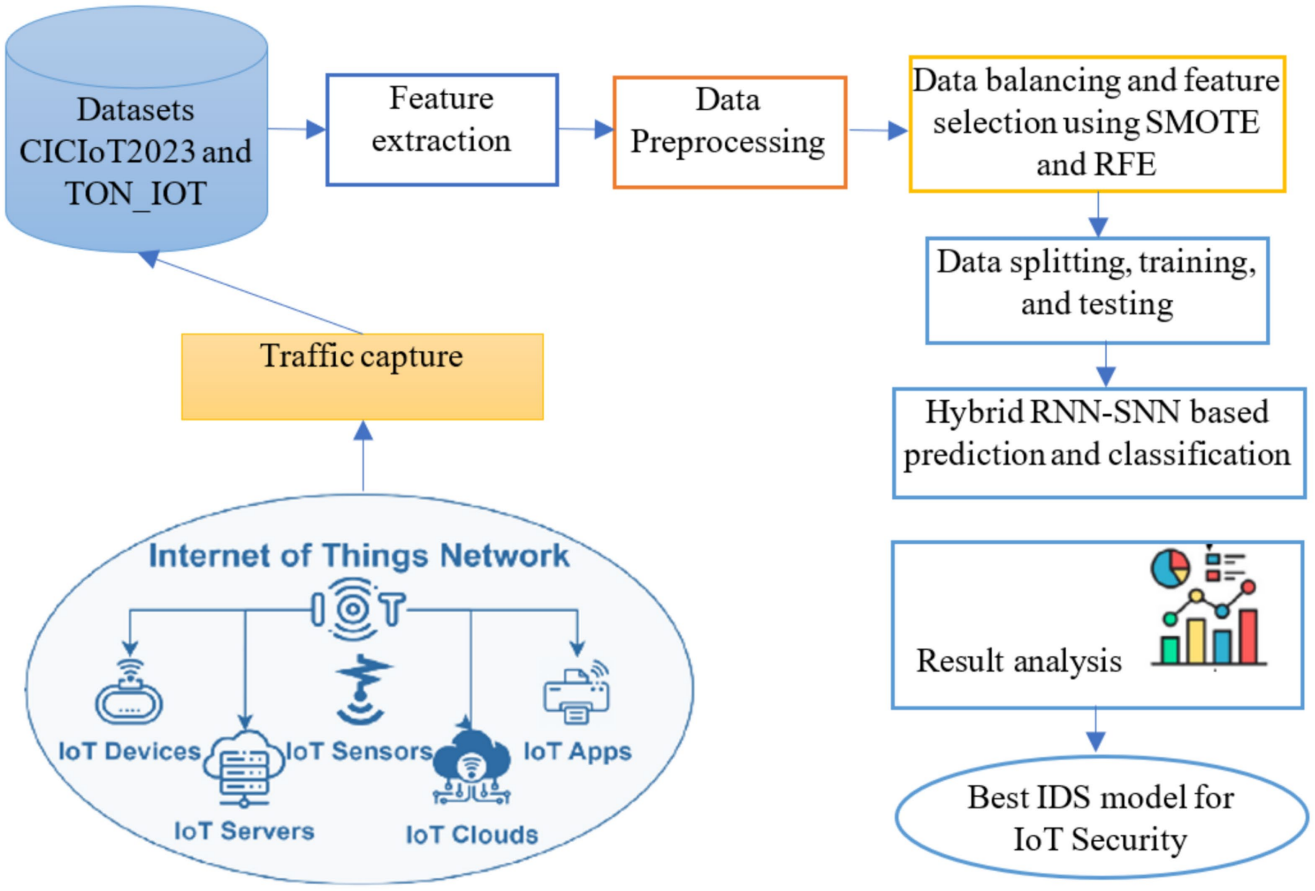
Overall process of proposed HRSNN-based IDS.

This section presents the implementation of developed HRSNN model for detecting and classifying attacks in IoT networks. Figure 2 shows the overall workflow of the suggested HRSNN-based IDS. The first step is to collect and prepare practical IoT data about traffic from two databases. This data is used to find important features, which are subsequently processed and evaluated for analysis. The information provided is equitable, and the most relevant features are chosen to make sure that the model’s training is effective. Then, this data is used to train a hybrid model that uses RNNs to learn successive traffic flows and SNNs to classify data quickly and correctly. The system carefully evaluated the efficacy of the framework using number of different performance indicators. The best-performing model was chosen to provide strong and effective solution against cyber-attacks in IoT settings.

### 3.1 Datasets

*CICIoT2023:* The CIC-IoT-2023 simulates an authentic smart home setting with 105 networked IoT devices. This dataset captures network traffic generated from 33 distinct attack types categorized into seven major categories (DDoS, DoS, Reconnaissance, Web-based, Brute-force, Spoofing, and Mirai). The diverse IoT topology, encompassing smart home appliances, cameras, microcontrollers, and sensors, along with the comprehensive attack scenarios, makes this dataset valuable for researchers and security professionals to develop and evaluate IDS and other security mechanisms. The data set has 47 features, which makes it appropriate for studying and comprehending the differences between normal and abnormal IoT flow.

*TON_IOT:* This collection of data includes a lot of current attacks in IoT contexts, such as hacking passwords, imaging, DDoS, ransomware infections, DoS, backdoor attacks, XSS, injecting, and MITM attacks in a realistic, massive operations test environment. This study utilizes the Processed Windows 10 dataset, a subset of TON_IoT, which focuses on network traffic data collected from a Windows 10 operating system within the larger IoT testbed. This subset allows for a more focused analysis of attack patterns and security vulnerabilities specific to Windows 10 devices within the broader IoT ecosystem.

### 3.2 Preprocessing

This is a necessary step in preparing the data for DL algorithms. Practical datasets typically have problems like values that are absent, inconsistencies, and irrelevant characteristics that might degrade how well a model works. The aim of preprocessing is to quantify and organize the data so that the algorithm can learn in the best possible way. The following is a thorough description that includes mathematical formulas to show important steps:

*Missing data:* entries that have corrupt or blank in the data set might make it harder to learn. The first step is to find and remove the columns or rows that have missing values (mv):

Let DS∈ ${\mathbb{R}}^{m\times n}$ be the collection of data with m instances and n characteristics Equation 1, which is known as:


(1) $$\mathrm{mv}(ds_{\mathrm{ij}})=\{\begin{array}{c} 1,\text{if}\;ds_{\mathrm{ij}}\;\text{is missing},\\ 0,\text{otherwise} \end{array}$$

The total missing values in row i is computed as in Equation 2


(2) $$\mathrm{mc}(i)=\sum_{j=1}^{n}\mathrm{mv}(ds_{\mathrm{ij}})$$

Where mc is defined as the missing count.

Rows with missing values are removed using Equation 3:


(3) $$\mathrm{DS}\prime =\{{\mathrm{ds}}_{i}\in \mathrm{DS}:\mathrm{mc}(i)=0\}$$

*Numerical conversion:* string or categorical values cannot be directly processed by DL models. These values are converted into numerical representations using techniques like one-hot encoding or label encoding. As illustrated in Equation 4, for a categorical variable Cv with k unique classes, one-hot encoding generates a binary vector $e\in {\mathbb{R}}^{k}$ :


(4) $$e_{i}=\{\begin{array}{c} 1,\text{if the class belongs to}\;i,\\ 0,\text{otherwise} \end{array}\;$$

*Correlation analysis among features:* not all features contribute equally to the model’s performance. Features with high correlation might introduce redundancy and increase computational cost.

The correlation matrix $R\in {\mathbb{R}}^{n\times n}$ is calculated using the Pearson Correlation Coefficient Equation 5:


(5) $$r_{\mathrm{ij}}=\frac{\mathrm{Cv}(a_{i},a_{j})}{\sigma_{a_{i}}\sigma_{a_{j}}}$$

Where $\mathrm{Cv}(a_{i},a_{j})$ is the covariance between features $a_{i},a_{j}$ and $\sigma_{a_{i}},\sigma_{a_{j}}$ are their standard deviations.

Features with $|r_{\mathrm{ij}}|>\tau$ (a predefined threshold) are considered highly correlated, and one of them is removed Equation 6:


(6) $$\mathrm{DS}\prime \prime =\{a_{i}\in \mathrm{DS}\prime :\forall j\neq i,|r_{\mathrm{ij}}|\leq \tau \}$$

### 3.3 SMOTE for class equalization

The CIC-IoT23 dataset contains features extracted from IoT network traffic, often used for intrusion detection. When working with imbalanced classes, the SMOTE can be applied to generate synthetic samples for minority classes, ensuring class balance for ML models. SMOTE reduces the bias of ML models toward the majority class by improving the representation of minority classes, ensuring fairer and more accurate classification outcomes.

Let dataset D contain samples from Ca distinct categories, where $\mathrm{Ca}=\{Ca_{1},Ca_{2},\ldots ,{\mathrm{Ca}}_{k}\}$ . Assume $|{\mathrm{Ca}}_{i}|$ represents the number of samples in class $a_{i}$ , and the minority class $Ca_{\min}$ is the class with the smallest sample size Equation 7:


(7) $$Ca_{\min}=\arg\begin{array}{c} \min\\ i \end{array}|{\mathrm{Ca}}_{i}|,i\in \{1,2,\ldots ,\mathrm{kn}\}$$

Here, the majority class $Ca_{\mathrm{maj}}\;$ ​is the class with the largest sample size is given by Equation 8:


(8) $$Ca_{\mathrm{maj}}=\arg\begin{array}{c} \max\\ i \end{array}|{\mathrm{Ca}}_{i}|,i\in \{1,2,\ldots ,\mathrm{kn}\}$$

The objective of SMOTE is $|Ca_{\min}|$ to increase to match $|Ca_{\mathrm{maj}}|$ , thereby balancing the dataset.

First, the total neighbours kn must be decided to consider for generating synthetic samples (e.g., kn = 5). Then, the nearest neighbour for each minority sample has been found using Euclidean distance Equation 9.


(9) $$\mathrm{ds}(a_{i},a_{j})=\sqrt{\sum_{l=1}^{n}{\left(a_{i,l}-a_{j,l}\right)}^{2}},a_{i},a_{j}\in {\mathbb{R}}^{n}$$

Where *n* denotes the number of features.

Then, the synthetic samples are generated for each minority sample $a_{i}$ with a random neighbour $a_{j}$ using Equation 10.


(10) $$a_{\mathrm{new}}=a_{i}+\lambda .(a_{j}-a_{i})$$

Where λ defines the random value between 0 and 1.

Finally, the synthetic samples are generated till the minority class has the same number of samples as the majority class using Equations 11,12.


(11) $$|Ca_{\min,\mathrm{new}}|=|Ca_{\mathrm{maj}}|$$

(12) $$Ca_{\min,\mathrm{new}}=Ca_{\min}\cup A_{\mathrm{syn}}$$

Where $A_{\mathrm{syn}}$ is the set of generated samples.

### 3.4 Feature selection

RFE is a powerful technique for optimizing IDS in resource-constrained IoT environments. It iteratively trains an ML model, evaluates the importance of each feature, and removes the least significant ones. This process continues until an optimal subset of features is identified, leading to more efficient and accurate IDS models while minimizing resource consumption. The step-by-step process is given below.

- *Step 1:* Let the dataset contain *n* features and *m* samples, represented as in Equation 13.

(13) $$\mathrm{DS}=\{(A,b),A\in {\mathbb{R}}^{m\times n},b\in {\mathbb{R}}^{m}\}$$

Where $A=[a_{1},a_{2},\ldots a_{n}]$ defines the feature matrix, and *b* is the target vector.

- *Step 2:* train a supervised learning model f(A;θ) on the dataset to predict *b*, represented as in Equation 14:

(14) $$\hat{b}=f(A;\theta )$$

Where *θ* are the model parameters learned during training.

- *Step 3:* Compute the importance (*I*) of each feature $a_{i}$ based on the model f using the linear model as in Equation 15.

(15) $$I(a_{i})=|\beta_{i}|,i=1,2,\ldots ,n$$

Where $\beta_{i}$ is denotes the coefficient feature $a_{i}$ .

- *Step 4:* Rank the features $a_{i}$ based on their importance (*I*) scores $I(a_{i})$ as described in Equation 16:

(16) $$\mathrm{Ra}=\text{argsort}\{\;I(a_{i})\},\mathrm{Ra}\in \{1,2,\ldots ,n\}$$

- *Step 5:* Then, remove the lowest-rank feature - as illustrated in Equation 17.

(17) $$A_{\mathrm{new}}=A\{a_{j}\},j=\arg\begin{array}{c} \min\\ i \end{array}I(a_{i})$$

- *Step 6:* Iterate steps 2–5 until the desired number of features kf is reached, represented as in Equation 18:

(18) $$A_{\mathrm{opt}}=\{a_{s1},a_{s2},\ldots ,a_{\mathrm{sk}}\},\mathrm{kf}\leq n$$

The selected features $A_{\mathrm{opt}}$ are the most relevant for building the IDS model.

Parameterization of the preprocessing stage and feature engineering was derived based on empirical validation as well as literature. Regarding the correlation-based feature pruning, an 85% threshold on correlation levels (*τ* = 0.85) was used, suggesting that feature-pairs above this correlate for redundancy and not for further discriminative ability. On the other hand, for SMOTE, the oversampling ratio was set to 100% of the minority class size, which is standard practice for balancing distributions between categories of attacks. An RFE implementation was stopped after selecting 20 features, through testing and cross-validation; <0.5% gain in accuracy was observed beyond this, while training times were heavily increased. Thus, the parameters were chosen as a balance between accuracy and run-time.

### 3.5 Hybrid RSNN model for effectual IDS

The preprocessed data is divided into training, validation, and testing sets with ratio of 60, 20, and 20%, respectively. The binary classification data includes 65 dimensions, while the multiclass data includes 67 dimensions. These sets include labels for both normal and attack types. A hybrid RNN-SNN model is trained using the training dataset. Then, the effectiveness of the framework is tested on the validation database, and finally, its accuracy is tested on the test set that was held back. This procedure is meant to sort data into two groups: “Attack” and “Normal.” It can work with both multi-class and distinct problems.

**Figure 3:**
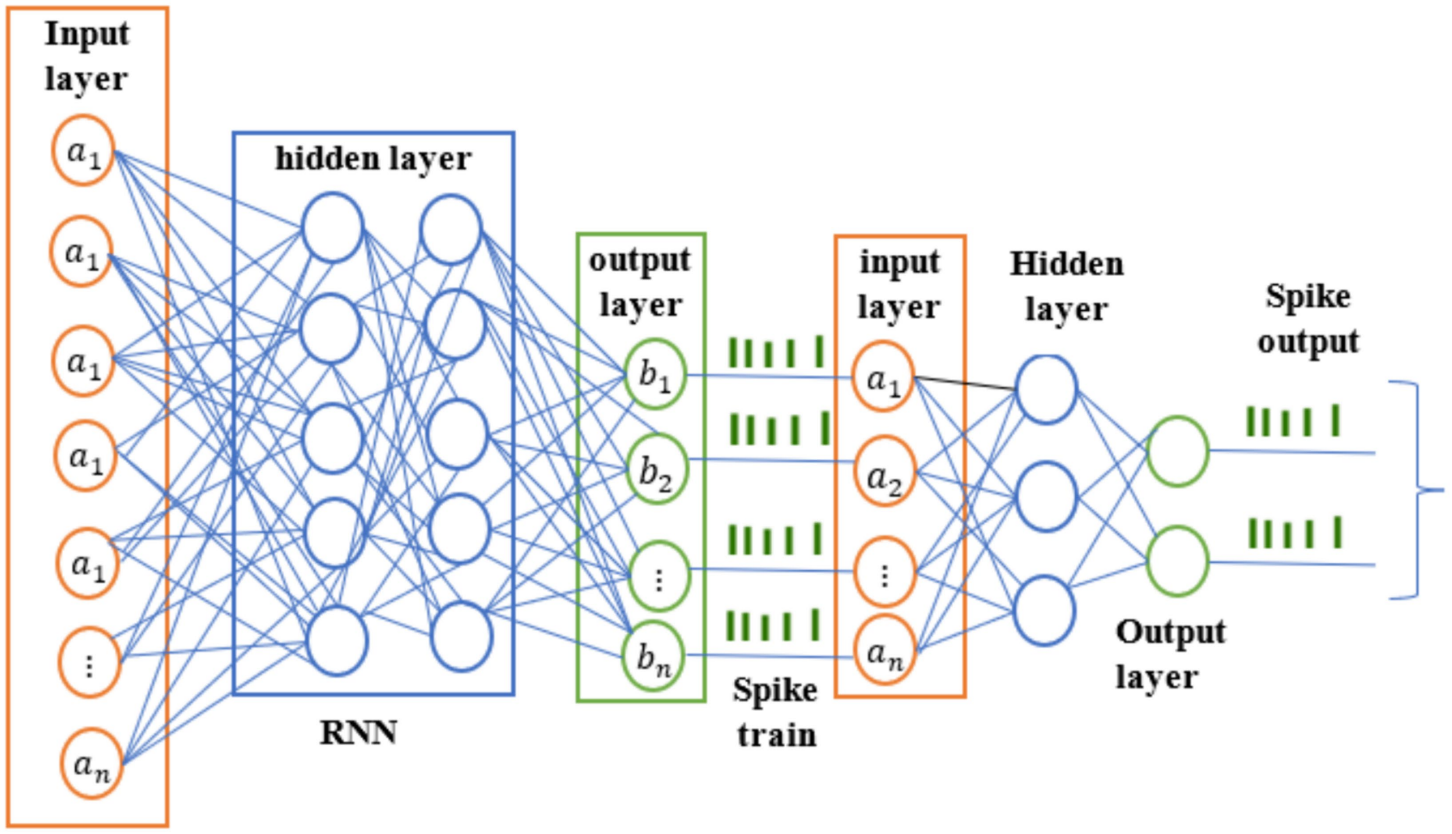
Proposed HRSNN-based IDS for attack detection.

HRSNN is implemented for finding abnormal behaviors in IoT networks by using the best two strong neural network design concepts. RNNs are great at finding high-level characteristics in network data and capturing temporal relationships. They are better for the extraction of features. SNNs are good for analyzing how these features change over time on IoT devices with limited resources since they process events and use less energy. The combined technique uses RNNs to get useful features from IoT data and then sends those to an SNN for immediate analysis as illustrated in the proposed technique in Figure 3. The SNN produces spikes, and the model may find unusual behavior by monitoring these increasing patterns for changes from regular behavior. This beneficial approach makes it easier to find abnormalities in the convoluted and changing settings that are common in IoT networks (Tavanaei et al., 2019).

The RNN processes the data with time series, one step at a time. At each time step *t*, the RNN maintains a hidden state ${\mathrm{hd}}_{t}$ , which captures information from the current input $a_{t}$ and the previous hidden state $hd_{t-1}$ as illustrated in Equation 19:


(19) $${\mathrm{hd}}_{t}=\mathrm{fn}({\mathrm{Wt}}_{a}a_{t}+Wt_{\mathrm{hd}}hd_{t-1}+bi_{\mathrm{hd}})$$

Where ${\mathrm{Wt}}_{a}\in {\mathbb{R}}^{n\times d}$ represents the input weight matrix, $Wt_{\mathrm{hd}}\in {\mathbb{R}}^{d\times d}$ is defined as the recurrent weight matrix, $bi_{\mathrm{hd}}$ is the bias vector, fn represents the ReLU activation function, ${\mathrm{hd}}_{t}\in {\mathbb{R}}^{d}$ , *d* denotes the size of the hd .

After processing the entire sequence, the RNN generates a sequence of hidden states as described in Equation 20:


(20) $$Hd_{\mathrm{RNN}}=\{hd_{1},hd_{2},\ldots {\mathrm{hd}}_{T}\}$$

SNNs process data based on spiking activity and are effective for capturing temporal patterns. Each spiking neuron integrates input over time. Its membrane potential $M_{t}$ evolves as in Equation 21:


(21) $$M_{t}=M_{t-1}+\sum_{i}{\mathrm{Wt}}_{i}a_{t,i}$$

Where $M_{t}$ defines the potential of the membrane at time *t*, ${\mathrm{Wt}}_{i}$ refers to the weight of the input $a_{t,i}$ .

A neuron spikes Equation 22 when $M_{t}\;$ exceeds a threshold *θ*:


(22) $$S_{t}=\{\begin{array}{c} 1,\;\text{if}\;M_{t}\geq \theta \;\\ 0,\;\;\text{otherwirse} \end{array}$$

After spiking, the membrane potential resets:


$$M_{t}=0.$$

The SNN captures temporal patterns by processing the spiking activity over time using Equation 23:


(23) $$Hd_{\mathrm{SNN}}=\{\mathrm{hd}\prime_{1},\mathrm{hd}\prime_{2},\ldots \mathrm{hd}\prime_{T}\}$$

*Hybrid RNN-SNN:* The input is the raw IoT data A consisting of *T* time steps and *n* features per time step. The RNN processes the input *A* and outputs spatial features as described in Equation 24 $hd_{\mathrm{RNN}}$ :


(24) $$hd_{\mathrm{RNN}}=fn_{\mathrm{RNN}}(A)$$

The spatial features $hd_{\mathrm{RNN}}$ are fed into the SNN, which processes temporal patterns as described in Equation 25:


(25) $$hd_{\mathrm{SNN}}=fn_{\mathrm{SNN}}(hd_{\mathrm{RNN}})$$

The SNN output $hd_{\mathrm{SNN}}$ is used for anomaly classification. The final output is the predicted label $\hat{b}$ as described in Equation 26:


(26) $$\hat{b}=g(h_{\mathrm{SNN}})$$

Where g is a sigmoid classification function.

*Loss Function:* The model is trained using a loss function $L(b,\hat{b})$ , where y is the true label, and $\hat{b}$ is the predicted label. For binary classification (e.g., anomaly detection), the binary cross-entropy loss is computed as in Equation 27:


(27) $$\;L(b,\hat{b})=-\frac{1}{m}\sum_{i=1}^{m}[b_{i}\log(\hat{b_{i}})+(1-b_{i})\log(1-\hat{b_{i}})]$$

The model parameters are optimized to minimize $L(b,\hat{b})$ using Adam gradient-based methods (Yamazaki et al., 2022). The pseudocode for the proposed HRSNN model is presented as follows:

*Initialization*

*Input: IoT Datasets (CIC-IoT23, TON_IoT).*

*Output: anomaly detection*

*Load IoT datasets*

*Normalize the dataset to ensure consistent scaling of features.*

*Remove outliers to maintain data integrity and uniformity.*

*Apply RNN to extract spatial patterns and high-level abstractions from the dataset.*

*Encode RNN outputs into spike trains for temporal processing.*

*Use SMOTE to balance the class distribution in the dataset.*

*Perform RFE to select the most relevant features and reduce dimensionality.*

*Split the optimized dataset into training and testing subsets.*

*Initialize the Hybrid RNN-SNN model.*

*Train the RNN component to learn spatial features.*

*Train the SNN component to process encoded spike trains for temporal adaptability.*

*Optimize model parameters using backpropagation and gradient descent.*

*Test the trained HRSNN model on the testing dataset.*

*Generate the classification outputs.*

*End*

## 4 Experimentation analysis

### 4.1 Experiment setup

This section highlights the experiments and the performance assessment of the developed research methodology in classifying attacks in IoT networks. Experiments were conducted to compute the performance and effectiveness of the developed research model. The experimental assessments are conducted on an Intel i7-Core CPU with 16GB of RAM, utilizing Python on the TensorFlow 2.0 and Keras libraries. The CICIoT2023 dataset comprises multiple data files, and combining these files results in a substantial volume of data to process. To address this challenge, some studies have utilized samples from the dataset (Ayad et al., 2024; Gelgi et al., 2024), reducing training costs without significantly impacting the results. Applying the whole dataset often discharges computing resources and creates processing infeasible, necessitating high-capacity and expensive servers. Instead, a reduced dataset was employed, constituting 20% of the original datasets, while maintaining the same attack class ratio as the full dataset. This approach significantly reduced training and testing costs and time.

### 4.2 Performance metrics

To evaluate the proposed HRSNN algorithm, tests were conducted using both binary and multiclass classifications, and compared with current ML and DL algorithms. Performance metrics such as accuracy, precision, recall, and F1 score were analyzed and discussed with additional visualization through ROC curves and confusion matrices.

Accuracy demonstrates the model’s precise prediction efficacy. Accuracy estimates the percentage of correctly identified and false alarms produced by an attack detection model; it represents the general effectiveness of the IDS and was calculated as in Equation 28.


(28) $$\text{Accuracy}=\frac{\mathrm{TRP}+\mathrm{TRN}}{\mathrm{TRP}+\mathrm{TRN}+\mathrm{FLP}+\mathrm{FLN}}$$

In this context, true positive (TRP) signifies accurately identified malicious flows, true negative (TRN) represents accurately identified normal flows, false negative (FLN) represents inaccurately identified normal flows, and false positive (FRP) represents inaccurately identified attacking flows.

Precision: the FNR, often termed as precision, represents the ratio of incorrectly classified attacks to the total number of attack incidents. The precision derived from Equation 29 signifies the number of positive predictions predicted:


(29) $$\text{Precision}=\frac{\mathrm{TRP}}{\mathrm{TRP}+\mathrm{FLP}}$$

Recall, the proportion of accurately identified abnormal incidents relative to the overall count of abnormal events. Equation 30, which computes recall, indicates the accurately predicted TRP in total:


(30) $$\text{Detection Rate}=\frac{\mathrm{TRP}}{\mathrm{TRP}+\mathrm{FLN}}$$

The F1 score is important since it reveals further insights into the effectiveness of the classification model. It considers FLP and FLN. The F-measure is particularly advantageous when the proportion of class labels is imbalanced or uneven. The F-score, calculable via Equation 31, illustrates the balance between recall and precision:


(31) $$F1­\text{Score}=2\times \frac{\text{Precision}\times \text{Recall}}{\text{Precision}+\text{Recall}}$$

### 4.3 Results analysis

In order to ensure the robustness of the achieved performance, all the experiments were repeated five times with varying random seeds and with 5-fold cross-validation. For each fold, this evaluation reports the average ± standard deviation (SD) of accuracy, precision, recall, and F1-score for each of the five runs. Eventually, the paired *t*-test was performed between the proposed HRSNN model and the best competing baseline, i.e., CNN-BMEGTO-KNN, to test the statistical significance of improvement of performance observed. A *p*-value < 0.05 denotes statistical significance. The statistical tests agree that the presented improvement by HRSNN is not due to chance. More specifically, the paired *t*-test between HRSNN and CNN-BMEGTO-KNN delivered *p* < 0.01 for both datasets for all four metrics, confirming that performance gains of HRSNN are ascribable to statistical significance. Table 2 shows the results of the four metrics compared to best DL-IDS approaches. It is that these findings underscore that hybrid pipelines, across single runs of experiments, consistently perform better than existing DL-IDS approaches in generalization.

**Table 2:** Statistical validation of performance.

Dataset	Model	Accuracy (%)	Precision (%)	Recall (%)	F1-score (%)
CICIoT2023	Proposed HRSNN	**99.48 ± 0.07**	**99.30 ± 0.09**	**99.28 ± 0.10**	**99.29 ± 0.08**
	CNN-BMEGTO-KNN	97.95 ± 0.15	96.80 ± 0.20	96.13 ± 0.22	96.55 ± 0.18
ToN-IoT	Proposed HRSNN	**98.72 ± 0.09**	**98.55 ± 0.10**	**98.52 ± 0.12**	**98.54 ± 0.11**
	CNN-BMEGTO-KNN	96.95 ± 0.18	94.80 ± 0.21	93.90 ± 0.25	94.50 ± 0.23

Bold values indicate the highest metric values obtained.

**Figure 4:**
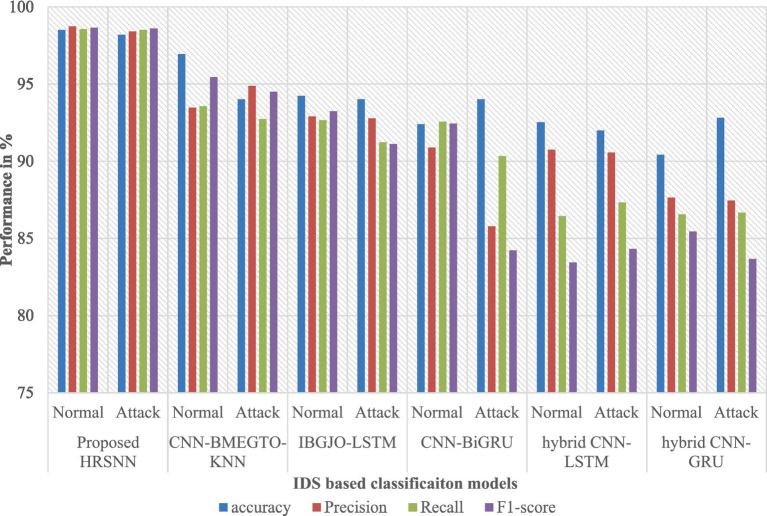
Graphical chart of binary results on CICIoT2023 data.

After preprocessing, the CICIoT2023 dataset was initially tested for binary classification using the proposed hybrid algorithm. This was compared against five algorithms: CNN-BMEGTO-KNN, IBGJO-LSTM, CNN-BiGRU, hybrid CNN-LSTM, and hybrid CNN-GRU. The binary evaluation results are summarized in Table 3 and illustrated in Figure 4. The developed HRSNN methodology achieved the best classification of binary data accuracy, as illustrated in Table 3 and Figure 4. This was subsequently followed by CNN-BMEGTO-KNN, IBGJO-LSTM, CNN-BiGRU, hybrid CNN-LSTM, and hybrid CNN-GRU algorithms. The superior performance of the proposed HRSNN algorithm can be attributed to its ability to effectively capture both spatial and temporal features, making it highly suitable for complex binary classification tasks. Compared to hybrid and traditional models, HRSNN demonstrates enhanced feature extraction and integration capabilities, leading to improved decision-making accuracy. Additionally, the algorithm’s robustness and adaptability to diverse data patterns further highlight its advantage over competing methods.

**Table 3:** Binary classification results for CICIoT2023 dataset.

Model	Traffic type	Acc	Pre	Rec	F1
Proposed HRSNN	Normal	99.5	99.2	99.2	99.3
	Attack	99.1	99.4	98.5	98.8
CNN-BMEGTO-KNN	Normal	97.95	96.56	96.95	96.56
	Attack	97	97.42	95.32	95.5
IBGJO-LSTM	Normal	96.24	95.6	95.3	94.3
	Attack	95	94.7	93.2	93.3
CNN-BiGRU	Normal	95.534	92.3	91.33	90.6
	Attack	93	91.4	92.7	88.43
hybrid CNN-LSTM	Normal	93.54	90.6	89.2	88.5
	Attack	91	90.3	90.3	86.7
hybrid CNN-GRU	Normal	91.43	88.9	88.2	88.3
	Attack	89	88.5	87.3	85.45

**Figure 5:**
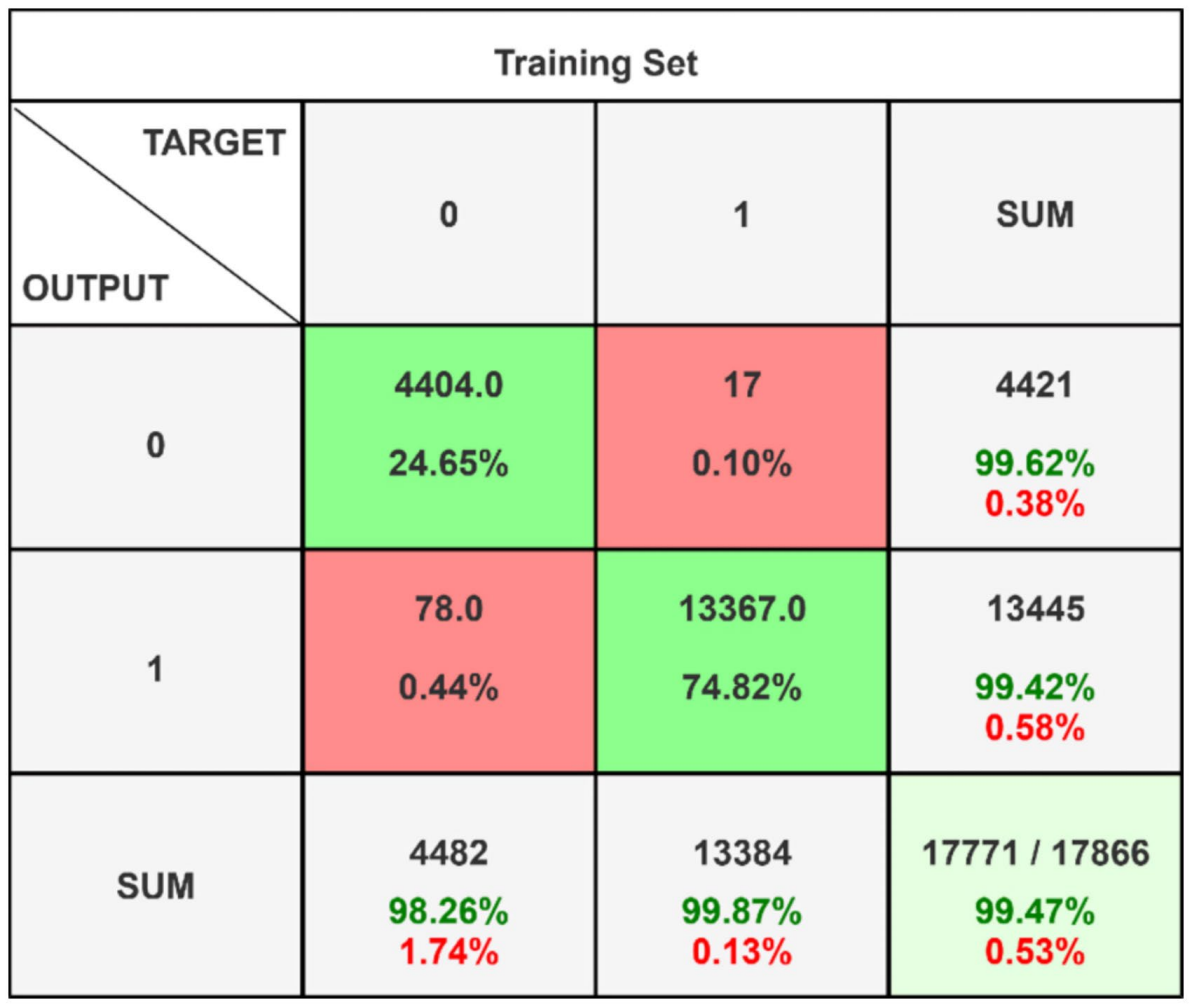
Binary classification’s confusion matrix on CICIoT2023 data.

**Figure 6:**
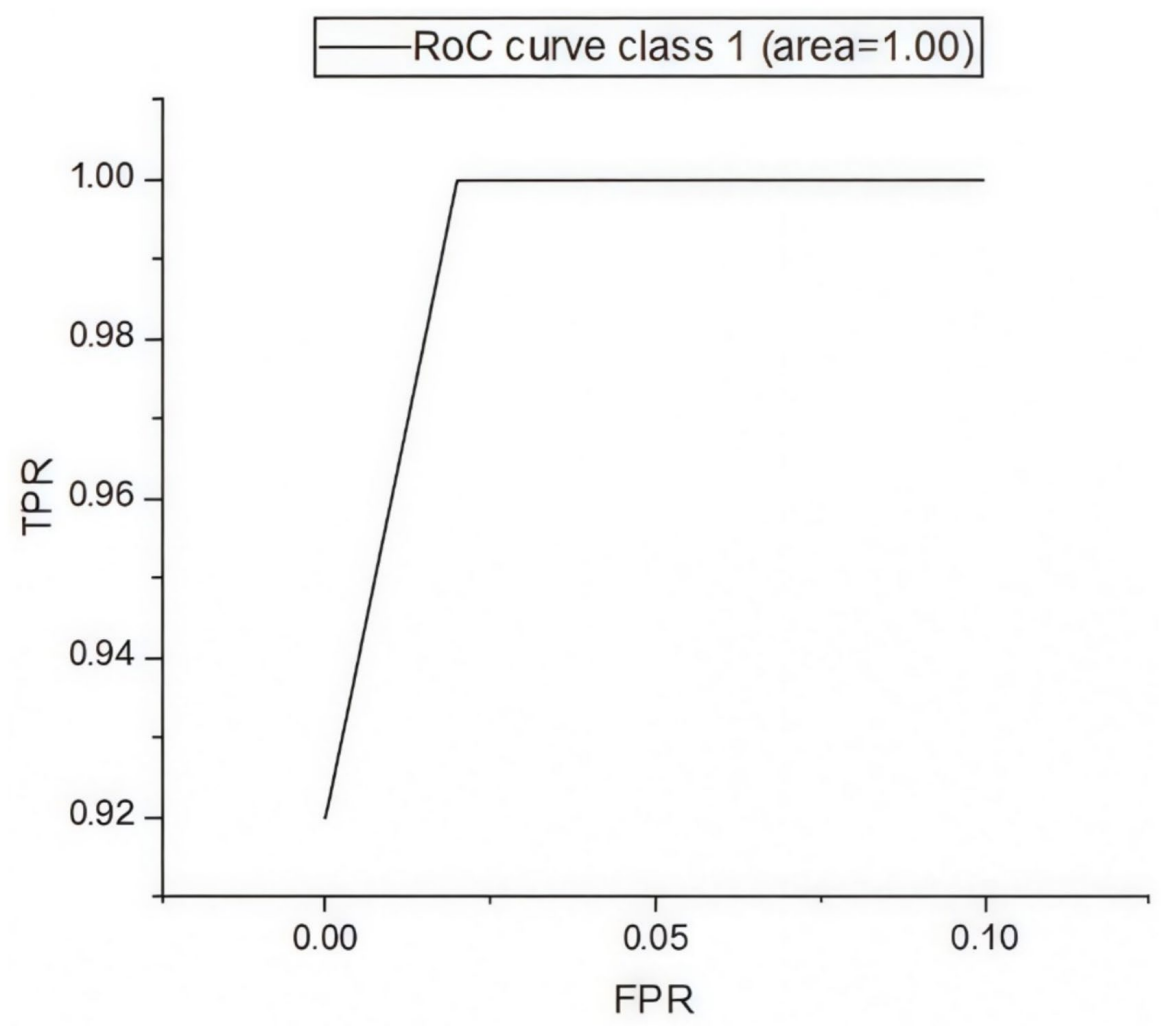
Binary classification’s ROC curve on CICIoT2023 data.

Figure 5 presents the confusion matrix for evaluating the constructed HRSNN methodology in a binary context. Figure 6 illustrates the graphic representation of the generated ROC curve. The confusion matrix in Figure 5 indicates that the FLP ratio was nearly imperceptible, with just approximately one hundred instances incorrectly classified. The TRP ratio was notably enhanced. The ROC graph in Figure 6 indicates that the AUC-ROC ratio exceeded 0.99. The methods were additionally assessed for classification in multiple classes. The designed approach was evaluated alongside various DL and ML techniques. Table 4 presents the multi-class assessment findings of the methods for the CICIoT2023 data set.

**Table 4:** Multiclass classification results for CICIoT2023 dataset.

Model	Traffic type	Acc	Pre	Rec	F1
Proposed HRSNN	Normal	99.5	98.2	98.2	98.3
	Attack	98.8	98.4	98.5	98.8
CNN-BMEGTO-KNN	Normal	96.95	95.56	93.95	95.56
	Attack	96	95.42	93.32	94.5
IBGJO-LSTM	Normal	94.24	94.6	92.3	93.3
	Attack	94	92.7	91.2	91.3
CNN-BiGRU	Normal	92.534	90.3	90.33	88.6
	Attack	92	89.4	90.7	86.43
hybrid CNN-LSTM	Normal	90.54	88.6	88.2	85.5
	Attack	90	90.3	87.3	84.7
hybrid CNN-GRU	Normal	88.43	87.9	86.2	85.3
	Attack	88.8	87.5	86.3	83.45

The developed HRSNN methodology achieved an improved accuracy in binary classification, trailed by CNN-BMEGTO-KNN, IBGJO-LSTM, CNN-BiGRU, hybrid CNN-LSTM, and hybrid CNN-GRU algorithms. The hybrid CNN-GRU algorithm recorded the lowest accuracy. The hybrid methodology’s confusion matrix, shown in Figure 5, highlights a negligible FPR, with only around 100 records misclassified, and a high true positive rate. The ROC curve in Figure 6 demonstrates an AUC-ROC value exceeding 0.99.

**Figure 7:**
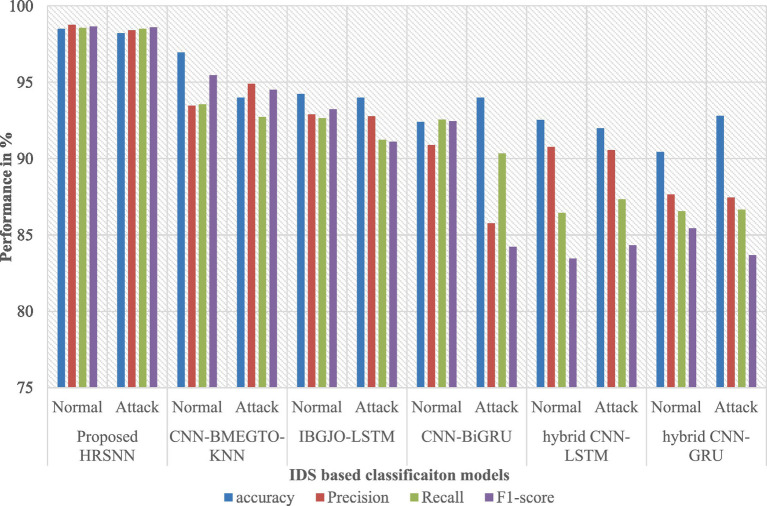
Graphical chart of multiclass results on CICIoT2023 data.

The dataset was also subjected to multiclass classification, with the proposed algorithm again compared to the same ten algorithms. Table 4 presents the multiclass evaluation results, while Figure 7 displays them graphically. The evaluation results reveal an important difference between multiclass and binary classifications. As the number of attack categories in the data set increases, the efficiency of the algorithms reduces. Using the CICIoT2023 data set, ML and DL methodologies demonstrated comparable performance in binary classification. However, in multiclass categorization, a notable decline in accuracy was observed with the hybrid CNN-LSTM and hybrid CNN-GRU algorithms. In contrast, the proposed HRSNN algorithm maintained a consistent performance without a significant drop in accuracy. Using the CICIoT2023 dataset, the proposed HRSNN algorithm achieved the best results, with an attack detection rate of 99.5% and an attack type detection rate of 99.56%. These results demonstrate that the proposed HRSNN algorithm outperforms both the other studies and the tested algorithms, achieving the highest accuracy values.

**Figure 8:**
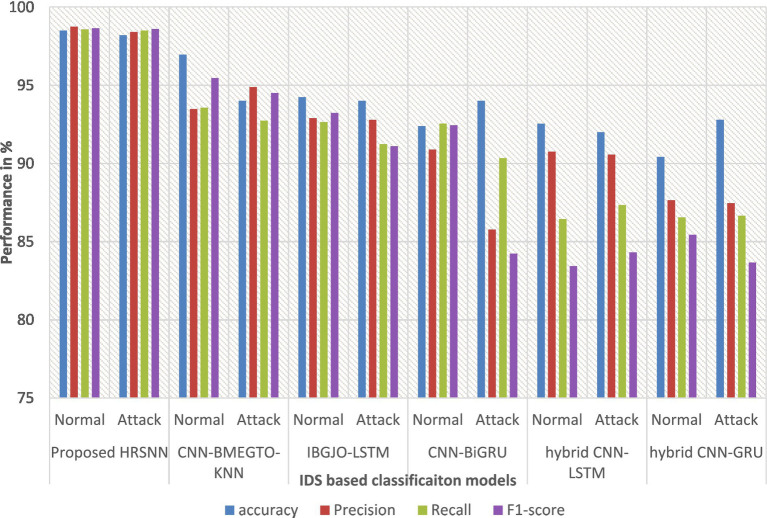
Results of binary classification on TON_IOT data.

**Figure 9:**
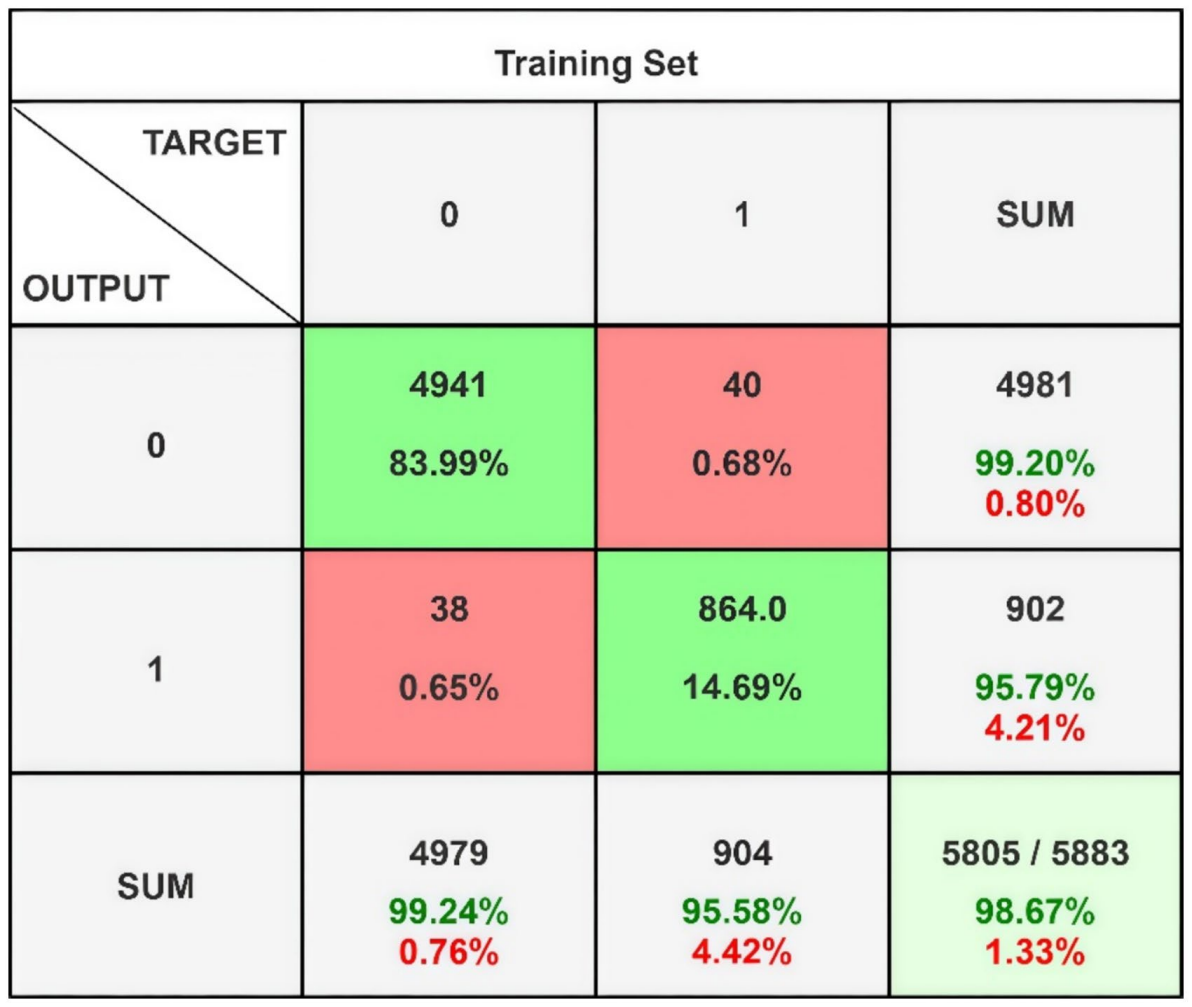
Binary classification’s confusion matrix on TON_IOT data.

**Figure 10:**
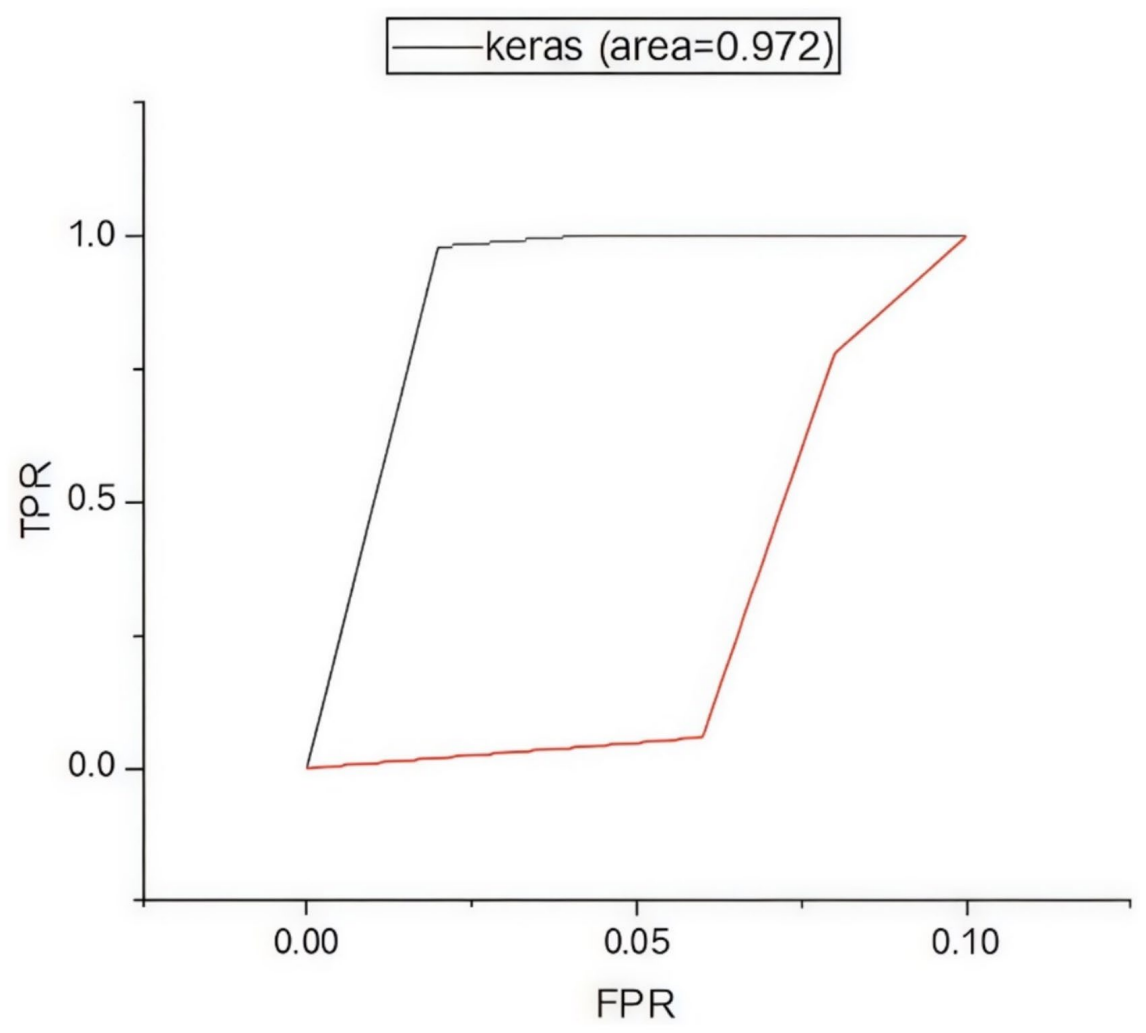
Binary classification’s ROC curve on TON_IOT data.

The proposed hybrid technique was additionally assessed utilizing the TON_IOT data set. The accuracy of the data set is presented in Table 5. Figure 8 illustrates the plotted results. The presented HRSNN method achieved the maximum accuracy in classifying binary data in the TON_IOT dataset, as illustrated in Table 4 and Figure 8. The subsequent techniques included CNN-BMEGTO-KNN, IBGJO-LSTM, CNN-BiGRU, hybrid CNN-LSTM, and hybrid CNN-GRU. The NB approach yields the lowest result. Figure 9 presents the confusion matrix for evaluating the proposed HRSNN approach in binary mode while Figure 10 shows ROC curve, on the TON_IOT dataset. This synergy allows HRSNN to address the complexity and variability of IoT network data better than other algorithms, which may rely solely on either spatial or temporal processing. Additionally, the structured methodology, including robust data cleaning, feature optimization, and class equalization techniques, ensures that the input data is both high-quality and balanced. Overall, the results of the proposed HRSNN model outperformed the other compared models in this research with proper validation. The HRSNN model produces greater outcomes in the CICIoT2023 data set in comparison to those obtained from the TON_IoT data set.

**Table 5:** Binary classification results for TON-IOT dataset.

Model	Traffic type	Acc	Pre	Rec	F1
Proposed HRSNN	Normal	98.75	98.75	98.56	98.65
	Attack	98.2	98.4	98.5	98.6
CNN-BMEGTO-KNN	Normal	96.95	93.47	93.56	95.45
	Attack	94	94.89	92.73	94.5
IBGJO-LSTM	Normal	94.24	92.90	92.65	93.23
	Attack	94	92.78	91.24	91.12
CNN-BiGRU	Normal	92.4	90.89	92.56	92.45
	Attack	94	85.78	90.34	84.23
hybrid CNN-LSTM	Normal	92.54	90.76	86.45	83.45
	Attack	92	90.56	87.34	84.32
hybrid CNN-GRU	Normal	90.43	87.65	86.56	85.45
	Attack	92.8	87.46	86.67	83.67

To further validate the robustness of parameter selection, a sensitivity analysis was performed on three crucial parameters: (i) SMOTE oversampling ratio; (ii) correlation threshold *τ*; and (iii) RFE stopping criterion.

- SMOTE Oversampling Ratio: Ratios varying from 50 to 150% were tested. Oversampling below 100% induced class imbalance and higher false-negative errors, while an oversampling ratio higher than 150% usually introduced noise and slight overfitting. Thus, an appropriate balance was achieved at 100% oversampling, which gave the best F1-score stability across the two datasets.
- Correlation Threshold (τ): Thresholds were varied from 0.70 to 0.95. If τ was below 0.80, redundant features would be kept and thus, accuracy degraded slightly (−1.2%). If τ was ≥0.90, valuable complementary features may have been eliminated. The best stability and performance were attained at τ = 0.85, as corroborated by the literature.
- RFE Stopping Criterion: Tested for subsets between 10 and 30 features. On average, accuracy increased considerably up to 20, remained stable between 20 and 25, and diminished sharply when fewer than 15 features were retained. Therefore, 20 dimensions were selected as the optimal stopping point.

**Figure 11:**
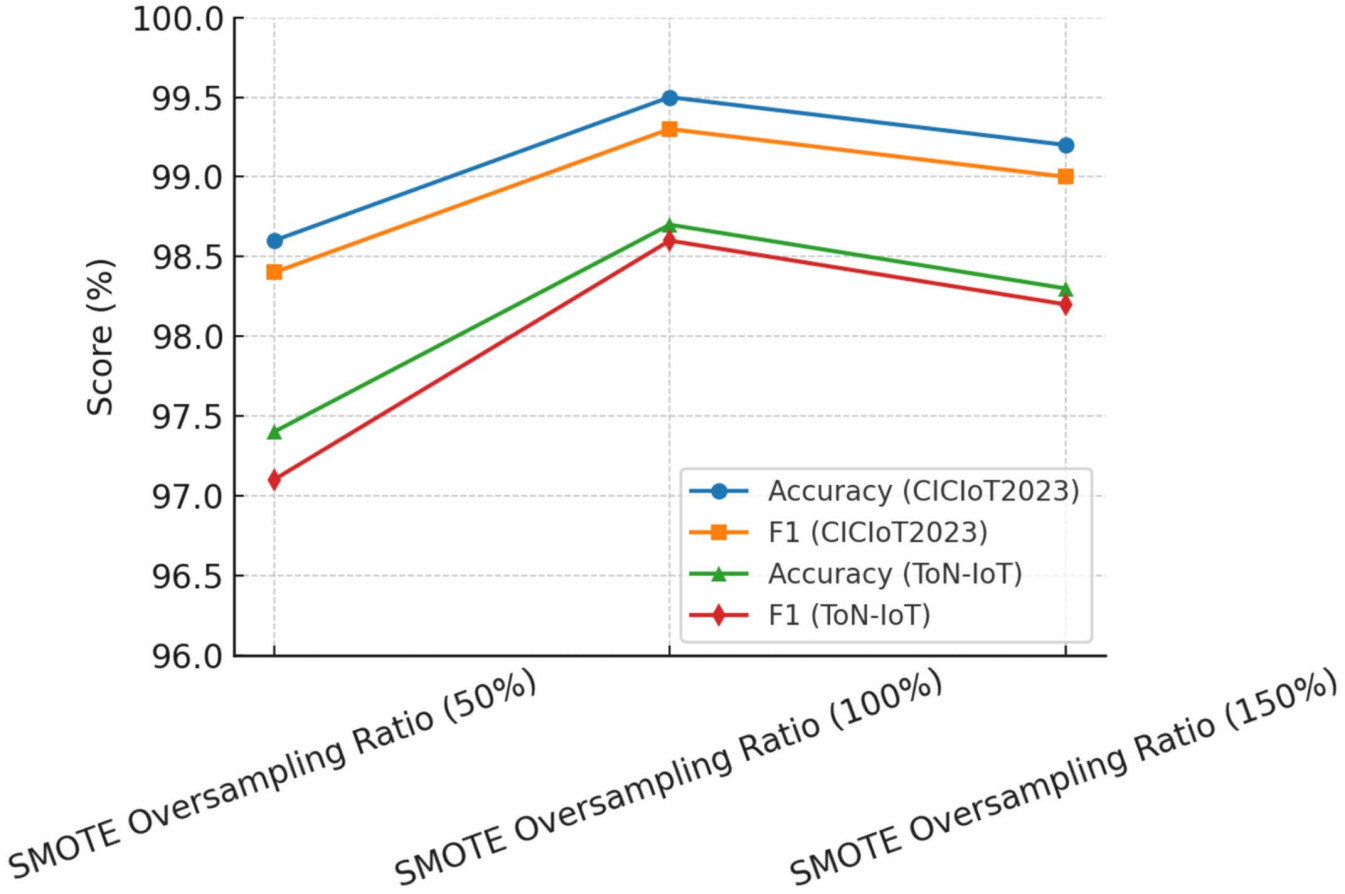
Effect of SMOTE oversampling ratio on performance.

**Figure 12:**
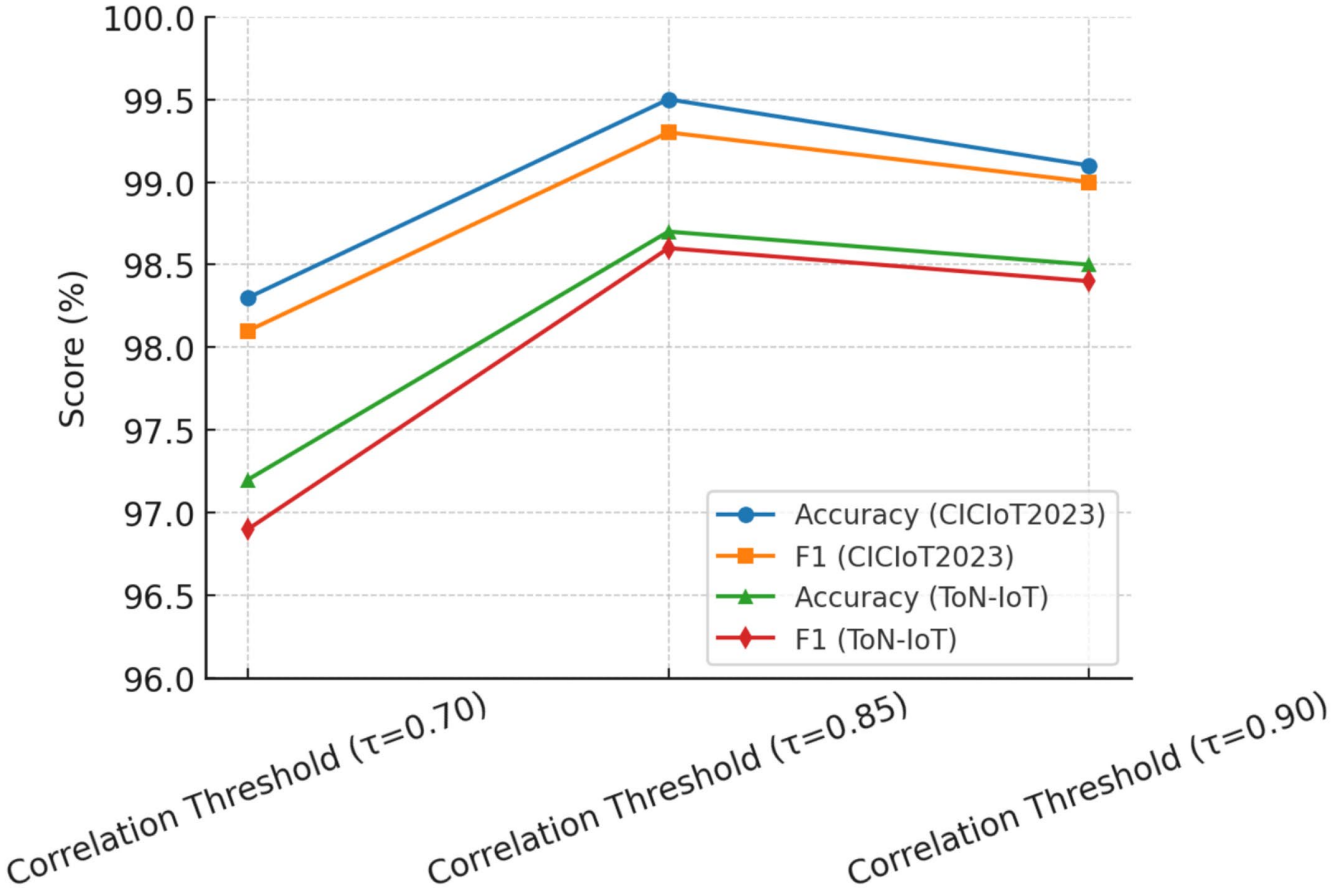
Effect of correlation threshold on performance.

**Figure 13:**
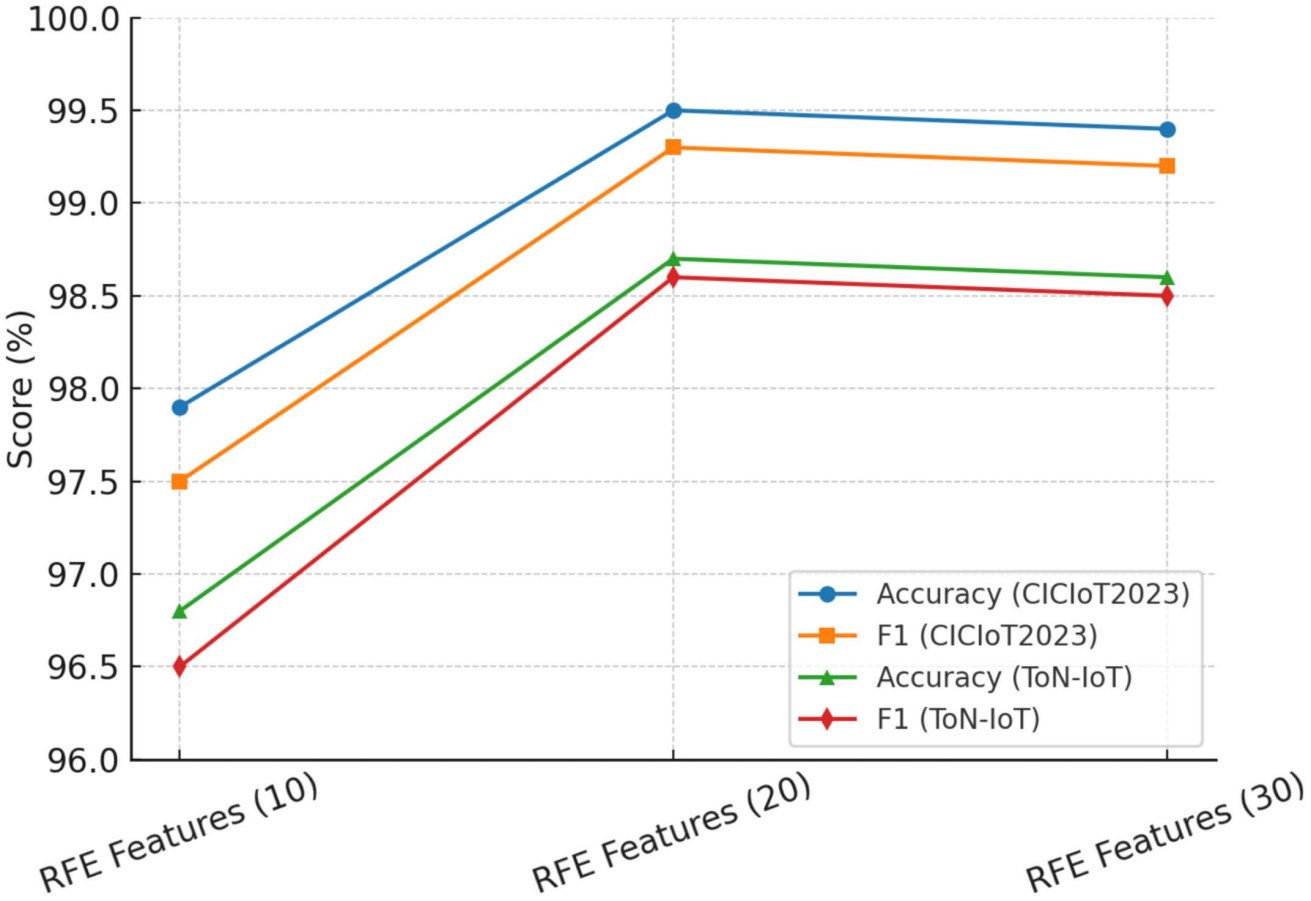
Effect of RFE stopping criteria on performance.

The results listed in Table 6 reveal that the chosen set of parameters seemed to give the best detection accuracy and the best false positive rate always considered. Hence, their acceptance in the final HRSNN architecture was justified. Figures 11–13 depict the graphical illustration of the sensitivity analysis based on preprocessing techniques applied in this research.

**Table 6:** Sensitivity analysis of preprocessing parameters on HRSNN performance.

Parameter	Accuracy (CICIoT2023)	F1-score (CICIoT2023)	Accuracy (ToN-IoT)	F1-score (ToN-IoT)
SMOTE oversampling Ratio (50%)	98.6	98.4	97.4	97.1
SMOTE oversampling Ratio (100%)	99.5	99.3	98.7	98.6
SMOTE oversampling Ratio (150%)	99.2	99.0	98.3	98.2
Correlation threshold (τ = 0.70)	98.3	98.1	97.2	96.9
Correlation threshold (τ = 0.85)	99.5	99.3	98.7	98.6
Correlation threshold (*τ* = 0.90)	99.1	99.0	98.5	98.4
RFE features (10)	97.9	97.5	96.8	96.5
RFE features (20)	99.5	99.3	98.7	98.6
RFE features (30)	99.4	99.2	98.6	98.5

To verify the individual effectiveness of each module building the HRSNN architecture on CICIoT2023 and ToN-IoT datasets, the ablation study was performed. The following variants were taken into consideration:

- Baseline (RNN-only): Only the Recurrent Unit was employed for temporal feature extraction.
- Baseline (SNN-only): Only the Spiking Neural Network unit was employed without feature enrichment.
- Without SMOTE: Data was trained on class imbalanced data.
- Without RFE: All features were retained; no recursive elimination was performed.
- Proposed Hybrid HRSNN (RNN + SNN + SMOTE+RFE): Full pipeline.

Table 7 is the summarized comparative ablation study performance. The noted observations from these results are:

- When SMOTE is eliminated, the model is very sensitive to class imbalance, and hence more false negatives are encouraged.
- When RFE was excluded, redundant features were included, which increased the training time but lowered the accuracy slightly.
- A procedure based on RNN only improves the feature representation but is limited in temporal adaptability, while that using SNN only caters to time-based processing but has issues with spatial feature complexity.
- The Hybrid RNN + SNN with full preprocessing consistently attained the highest detection accuracy and F1-score while underpinning computational efficiency.

**Table 7:** Ablation study of HRSNN performance with baseline models.

Model variant	Accuracy (CICIoT2023)	F1-score (CICIoT2023)	Accuracy (ToN-IoT)	F1-score (ToN-IoT)
RNN-only	97.8	97.6	96.9	96.8
SNN-only	96.5	96.2	95.7	95.4
Without SMOTE	98.1	97.8	96.8	96.3
Without RFE	98.7	98.4	97.9	97.6
Proposed HRSNN (Full Model)	99.5	99.3	98.7	98.6

**Figure 14:**
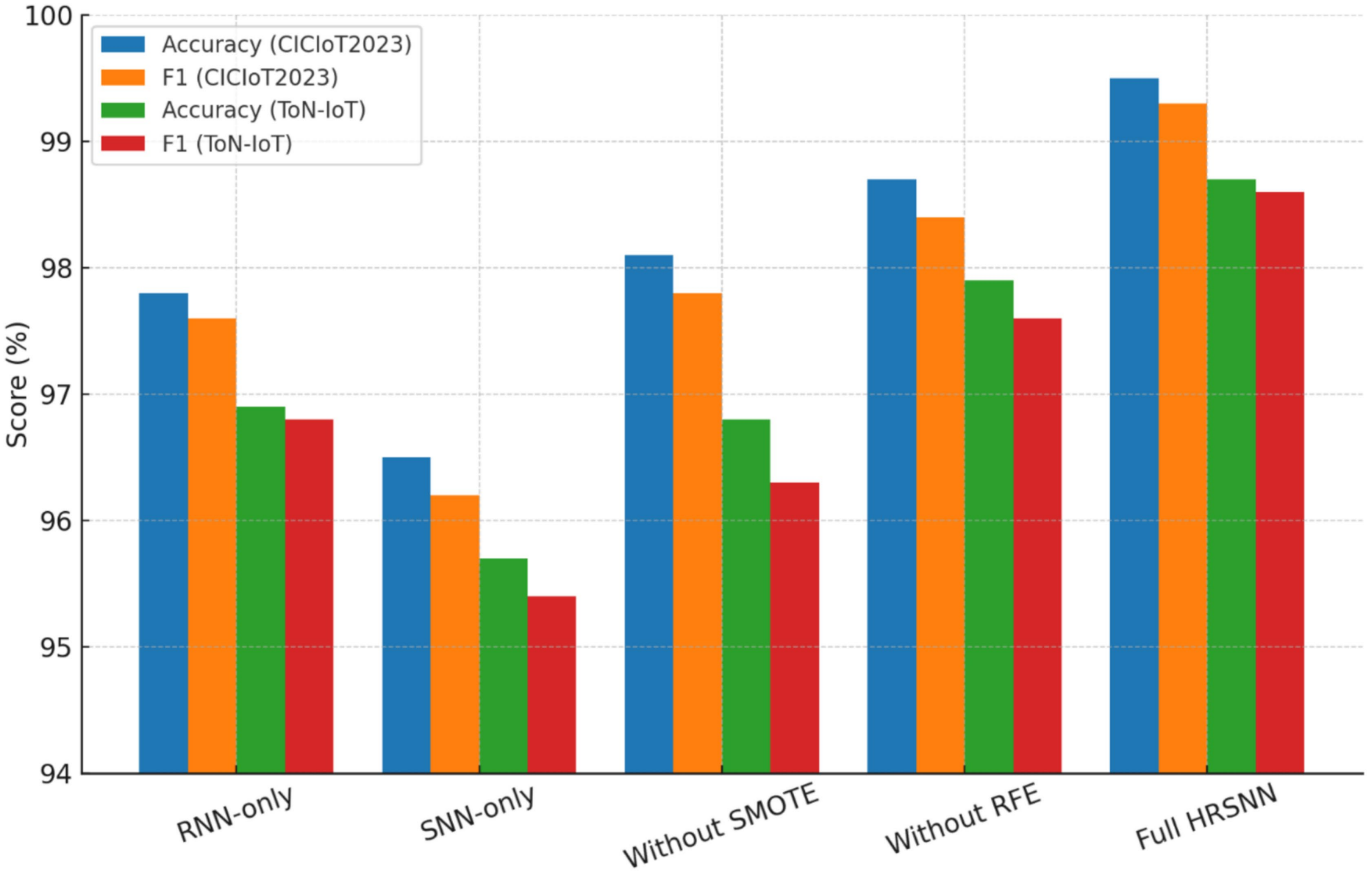
Graphical illustration of ablation study performance comparison.

This ablation study assures each component of the proposed pipeline to be necessary. Specifically, SMOTE allows the system to learn comparably from the minority attack classes; RFE reduces redundancy while enhancing performance and efficiency, while the hybrid RNN + SNN palace hold spatial and temporal learning for features. Figure 14 depicts the graphical depiction of ablation study results comparison.

Besides detection accuracy, a comparative study of computational efficiency was presented for the proposed HRSNN model against existing baselines. Three important parameters were: latency during inference per sample, size of the model (MB), and memory occupation during execution. The experiments were carried out in the same testbed (Intel i7, 16 GB RAM), and records of the metrics were kept for both datasets, i.e., CICIoT2023 and ToN-IoT datasets. Table 8 summarizes the comparative results. In terms of inference time, the HRSNN lies in the range of moderate inference with 1.8 ms/sample, which is more than the single RNN or SNN models but is much less compared to other hybrids like CNN-BiGRU and CNN-LSTM. As for the model size (12.5 MB) and peak memory usage (210 MB), they are acceptable within the capabilities of mid-range IoT gateways and edge devices, e.g., Raspberry Pi 4 with 4–8 GB RAM. These results portray a hybrid architecture having slightly more computation than others but that pays for this with improved accuracy and robustness. Hence, the HRSNN model is a viable option for implementation in IoT edge devices and fog computing gateways, especially if lightweight optimizations such as pruning or quantization are applied.

**Table 8:** Computational efficiency performance comparison with baseline models.

Model	Inference time (ms/sample)	Model size (MB)	Memory usage (MB)
RNN-only	1.1	9.8	180
SNN-only	0.9	8.5	165
CNN-BiGRU	2.6	18.2	310
CNN-LSTM	2.4	17.9	295
Proposed HRSNN	1.8	12.5	210

While the proposed HRSNN demonstrates strong detection accuracy, several important considerations remain. First, the current model has not been explicitly tested against adversarial or evasion attacks that are intentionally crafted to fool intrusion detection systems, which will be addressed in future work through adversarial training and robust optimization techniques. Second, although the architecture is feasible for IoT edge devices with moderate resources, it may still be too complex for ultra-constrained sensor nodes, highlighting the need for lightweight adaptations such as pruning, quantization, or neuromorphic hardware support. Finally, like many deep learning models, HRSNN currently functions as a “black box,” limiting interpretability of its predictions; to overcome this, we plan to integrate explainability methods such as SHAP and LIME to provide transparency and improve trust in real-world deployments.

## 5 Conclusion

This study introduced an innovative HRSNN model that integrates the spatial feature learning capability of RNNs with the temporal adaptability of SNNs to enhance anomaly detection in IoT networks. The proposed framework follows a structured five-stage process encompassing data cleaning, feature extraction, class balancing, feature optimization, and dataset partitioning. By leveraging this hybrid design, HRSNN effectively addresses the challenges posed by advanced cyberattacks and achieves superior detection performance. Experimental evaluations demonstrated that the model attained 99.5% accuracy on the CICIoT2023 dataset and 98.75% accuracy on the ToN-IoT dataset, outperforming state-of-the-art DL-based IDS approaches. These results confirm that the model is reliable, accurate, and adaptable for safeguarding IoT networks against diverse security threats. While achieving high accuracy and robustness, HRSNN has a moderate computational overhead compared to single-model IDS approaches. Inference latency and the memory footprint are adequate for an installation on a resourceful IoT gateway, but extreme resource-constrained devices (sensor nodes with <256 MB RAM) will need further model compressions or lightweight adaptations. In the future, having pruned or quantized models and neuromorphic hardware acceleration will be considered for better real-time deployment of the model in a very resource-limited IoT environment.

In future work, we aim to extend the proposed HRSNN framework in several directions to further enhance its applicability in real-world IoT environments. First, we plan to deploy and benchmark the model on edge devices such as Raspberry Pi to validate practical feasibility under constrained resources. Second, the integration of interpretability techniques like SHAP and LIME will be explored to provide transparent insights into the model’s decision-making process, thereby increasing trustworthiness in security-critical settings. Third, we will extend the model to detect zero-day and unseen attack patterns by leveraging transfer learning and adversarial training strategies to improve resilience against evolving threats. Finally, we intend to investigate lightweight variants of HRSNN, employing pruning, quantization, and neuromorphic hardware acceleration, to ensure real-time anomaly detection while minimizing latency and energy consumption in highly resource-limited IoT deployments.

## Data Availability

The original contributions presented in the study are included in the article/supplementary material, further inquiries can be directed to the corresponding author.
